# Supplementary material for: Multilocus Phylogeny and Characterization of Five Undescribed Aquatic Carnivorous Fungi (Orbiliomycetes)
Source: J Fungi (Basel). 2024 Jan 20;10(1):81. doi: 10.3390/jof10010081 (PMC10817524; doi:10.3390/jof10010081)
Supplement: Supplementary file 1 [file jof-10-00081-s001.zip › jof-2789001-supplementary.pdf]

Table S1. GenBank accession numbers involved in this study. Ex-type strains are in bold. The newly generated sequences are indicated in blue.

Taxon	Strain Number	GenBank accession Number		
		ITS	TEF1-α	RPB2
Arthrobotrys amerospora	**CBS 268.83**	**NR_159625**	——	——
A. anomala	YNWS02-5-1	AY773451	AY773393	AY773422
A. arthrobotryoides	AOAC	MF926580	——	——
A. blastospora	**CGMCC 3.20940**	**OQ332405**	**OQ341651**	**OQ341649**
A. botryospora	**CBS 321.83**	**NR_159626**	——	——
A. cibiensis	**DLUCC 109**	**OR880379**	**OR882792**	**OR882797**
A. cibiensis	**EY10**	**OR902195**	**OR882787**	**OR882802**
A. cladodes	1.03514	MH179793	MH179616	MH179893
A. clavispora	**CBS 545.63**	**MH858353**	——	——
A. conoides	670	AY773455	AY773397	AY773426
A. cookedickinson	YMF 1.00024	MF948393	MF948550	MF948474
A. cystosporia	CBS 439.54	MH857384	——	——
A. dendroides	YMF 1.00010	MF948388	MF948545	MF948469
A. dianchiensis	1.00571	MH179720	——	MH179826
A. elegans	1.00027	MH179688	——	MH179797
A. eryuanensis	**CGMCC 3.19715**	**MT612105**	**OM850307**	**OM850301**
A. eudermata	SDT24	AY773465	AY773407	AY773436
A. flagrans	1.01471	MH179741	MH179583	MH179845
A. gampsospora	CBS 127.83	U51960	——	——
A. globospora	1.00537	MH179706	MH179562	MH179814
A. gongshanensis	**CGMCC 3.23753**	**OM801277**	**OM809162**	**OM809163**
A. guizhouensis	YMF 1.00014	MF948390	MF948547	MF948471
A. heihuiensis	**DLUCC 108-1**	**OR880378**	**OR882791**	**OR882796**
A. heihuiensis	**Y710**	**OR902194**	**OR882786**	**OR882801**
A. hengjiangensis	**CGMCC 3.24983**	**OQ946587**	**OQ989312**	**OQ989302**
A. hyrcanus	**IRAN 3650C**	**MH367058**	**OP351540**	——
A. indica	YMF 1.01845	KT932086	——	——
A. iridis	521	AY773452	AY773394	AY773423
A. janus	85-1	AY773459	AY773401	AY773430
A. javanica	105	EU977514	——	——
A. jindingensis	**CGMCC 3.20895**	**OP236810**	**OP272511**	**OP272515**
A. jinpingensis	**CGMCC 3.20896**	**OM855569**	**OM850311**	**OM850305**
A. jinshaensis	**DLUCC 133**	**OR880381**	**OR882794**	**OR882799**
A. jinshaensis	**MA142**	**OR902197**	**OR882789**	**OR882804**
A. koreensis	C45	JF304780	——	——
A. lanpingensis	**CGMCC 3.20998**	**OM855566**	**OM850308**	**OM850302**
A. latispora	H.B. 8952	MK493125	——	——

A. longiphora	1.00538	MH179707	——	MH179815
A. lunzhangensis	**CGMCC 3.20941**	**OK643973**	**OM621809**	**OM621810**
A. luquanensis	**CGMCC 3.20894**	**OM855567**	**OM850309**	**OM850303**
A. mangrovispora	MGDW17	EU573354	——	——
A. megalospora	TWF800	MN013995	——	——
A. microscaphoides	YMF 1.00028	MF948395	MF948552	MF948476
A. multiformis	**CBS 773.84**	**MH861834**	——	——
A. musiformis	SQ77-1	AY773469	AY773411	AY773440
A. musiformis	1.03481	MH179783	MH179607	MH179883
A. nonseptata	**YMF 1.01852**	**FJ185261**	——	——
A. obovata	**YMF 1.00011**	**MF948389**	**MF948546**	**MF948470**
A. oligospora	920	AY773462	AY773404	AY773433
A. paucispora	ATCC 96704	EF445991	——	——
A. polycephala	1.01888	MH179760	MH179592	MH179862
A. pseudoclavata	1130	AY773446	AY773388	AY773417
A. psychrophila	1.01412	MH179727	MH179578	MH179832
A. pyriformis	YNWS02-3-1	AY773450	AY773392	AY773421
A. reticulata	CBS 550.63	MH858355	——	——
A. robusta	nefuA4	MZ326655	——	——
A. salina	SF 0459	KP036623	——	——
A. scaphoides	1.01442	MH179732	MH179580	MH179836
A. shizishanna	YMF 1.00022	MF948392	MF948549	MF948473
A. shuifuensis	**CGMCC 3.19716**	**MT612334**	**OM850306**	**OM850300**
A. sinensis	105-1	AY773445	AY773387	AY773416
A. sphaeroides	1.01410	MH179726	MH179577	MH179831
A. superba	127	EU977558	——	——
A. thaumasia	917	AY773461	AY773403	AY773432
A. tongdianensis	**CGMCC 3.20942**	**OP236809**	**OP272509**	**OP272513**
A. vermicola	629	AY773454	AY773396	AY773425
A. weixiensis	**CGMCC 3.24984**	**OQ946585**	**OQ989310**	**OQ989300**
A. xiangyunensis	**YXY10-1**	**MK537299**	——	——
A. yangbiensis	**DLUCC 36-1**	**OR880382**	**OR882795**	**OR882800**
A. yangbiensis	**Y678**	**OR902198**	**OR882790**	**OR882805**
A. yangjiangensis	**DLUCC 124**	**OR880380**	**OR882793**	**OR882798**
A. yangjiangensis	**YB19**	**OR902196**	**OR882788**	**OR882803**
A. yunnanensis	**YMF 1.00593**	**AY50993**	——	——
A. zhaoyangensis	**CGMCC 3.20944**	**OM855568**	**OM850310**	**OM850304**
Dactylellina cangshanensis	**CGMCC 3.19714**	**MK372062**	**MN915115**	**MN915114**
Da. copepodii	**CBS 487.90**	**U51964**	**DQ999835**	**DQ999816**
Da. mammillata	**CBS 229.54**	**AY902794**	**DQ999843**	**DQ999817**
Da. yushanensis	**CGMCC 3.19713**	**MK372061**	**MN915113**	**MN915112**
Drechslerella coelobrocha	FWY03-25-1	AY773464	AY773406	AY773435

Dr. dactyloides	expo-5	AY773463	AY773405	AY773434
Dr. daliensis	**CGMCC 3.20131**	**MT592896**	**OK556701**	**OK638157**
Dr. heterospora	YMF 1.00550	MF948400	MF948554	MF948480
Dr. stenobrocha	YNWS02-9-1	AY773460	AY773402	AY773431
Dr. xiaguanensis	**CGMCC 3.20132**	**MT592900**	**OK556699**	**OK638159**
Orbilia jesu-laurae	**LQ59a**	**MN816816**	**——**	**——**
O. tonghaiensis	**YMF 1.03006**	**NR_172397**	**MF948570**	**MF948496**
Vermispora fusarina	YXJ02-13-5	AY773447	AY773389	AY773418
V. leguminacea	CGMCC 6.0291	NR_173249	——	——
